# Methylglyoxal as a Convergent Mediator of Diabetic Complications: Generation, Protein Targets, Tissue Distribution, and Therapeutic Reduction—A Clinically Oriented Mechanistic Synthesis

**DOI:** 10.3390/biom16081104

**Published:** 2026-07-29

**Authors:** Enrique C. Fernandez

**Affiliations:** HCA Florida Kendall Hospital, Miami, FL 33175, USA; fernandezmde@gmail.com

**Keywords:** methylglyoxal, MGO, MG-H1, glyoxalase, GLO1, reactive dicarbonyl, RAGE, type 2 diabetes mellitus, diabetic complications, clinical biomarkers, mechanistic review

## Abstract

Methylglyoxal (MGO), a highly reactive 1,2-dicarbonyl, is generated by all three principal pathways of advanced glycation end product (AGE) synthesis in type 2 diabetes mellitus (T2DM)—the Hodge, Namiki, and Wolff pathways—and by the non-enzymatic degradation of glycolytic triose phosphates. It is the principal substrate of the glutathione-dependent GLO1/GLO2 glyoxalase system and the main source of the hydroimidazolone-1 (MG-H1) adduct, and it directly modifies intracellular proteins across multiple tissues. This clinically oriented narrative review synthesizes dicarbonyl chemistry, glyoxalase, and AGE adduct research to propose—as a hypothesis-generating schema rather than established biology—that MGO functions as a convergent biochemical node in diabetic complications. We examine MGO generation across the four input routes, its preferential modification of arginine and lysine residues, the correspondence between tissue MGO accumulation and complication distribution, glyoxalase-mediated clearance, and therapeutic strategies. We further propose that routine parameters such as gamma-glutamyl transferase and red cell distribution width may serve as accessible proxies for MGO burden, with the explicit caveat that these mappings require prospective validation and do not constitute a validated clinical instrument.

## 1. Introduction

The pathogenesis of diabetic complications has been explained through several partially independent biochemical mechanisms: the polyol pathway, the protein kinase C pathway, the hexosamine pathway, the AGE synthesis pathways, and oxidative stress [1]. Each mechanism has its supporting experimental evidence, its candidate inhibitor pharmacology, and its body of clinical research. Each explains some complications in some experimental systems. However, clinical trials targeting individual mechanisms—aldose reductase inhibitors for the polyol pathway, PKC-β inhibitors, and AGE cross-link breakers—have generally not translated preclinical efficacy into robust human clinical benefit [2,3]. Brownlee proposed that the multiple mechanisms share a common upstream driver in mitochondrial superoxide overproduction, providing a unifying account of why pathway-specific interventions might fail [1].

A complementary framing, developed in this manuscript, focuses on the convergence of these mechanisms onto a single reactive intermediate: methylglyoxal (MGO). MGO is generated by all three classical AGE synthesis pathways (Hodge, Namiki, Wolff), independently produced by the non-enzymatic degradation of triose phosphate intermediates of glycolysis, cleared predominantly by a single enzyme system (the GLO1/GLO2 glyoxalase axis), and responsible for the formation of the major AGE adducts that activate the receptor for advanced glycation end products (RAGE). It also directly modifies intracellular proteins—including tricarboxylic acid (TCA) cycle enzymes and structural proteins of the erythrocyte membrane and extracellular matrix—through covalent adduct formation [4].

This synthesis is not a claim of novelty regarding MGO biology, which has been extensively characterized in the dicarbonyl chemistry and glyoxalase literature for several decades [3]. Rather, this paper synthesizes the established literature with the goal of articulating MGO’s convergent role in a manner accessible to clinical interpretation.

**Relationship to prior work and statement of novelty.** The author has previously reviewed the three classical AGE synthesis pathways (Hodge, Namiki, and Wolff) as an organizing subject in their own right [4]. To avoid duplication, detailed characterization of those individual pathways is not repeated here; the present manuscript instead treats their *convergence*—together with the glycolytic triose phosphate route—onto a single reactive intermediate, methylglyoxal, and develops the downstream consequences of that convergence, aspects that were not the focus of their earlier work: the tissue-specific pattern of MGO protein modification (Section 3), the correspondence between tissue MGO accumulation and the clinical distribution of complications (Section 4), and the therapeutic and biomarker implications of treating MGO as a shared node (Section 6, Section 7 and Section 8). The proposal that routine parameters such as gamma-glutamyl transferase (GGT) and red cell distribution width (RDW) may be interpreted in relation to MGO biology is not original to this manuscript; both parameters have established observational research in diabetes (developed in Section 7), and the only incremental step taken here is to frame those established associations within an MGO-centered mechanistic account, explicitly as a hypothesis-generating schema rather than a validated clinical algorithm.

**Convergent MGO framing relative to established mechanisms.** The proposal that MGO acts as a convergent node should be situated critically against, not substituted for, the established pathogenic mechanisms. The convergence framing is partial rather than comprehensive: the polyol and hexosamine pathways have outputs and signaling consequences that are independent of MGO; PKC activation is driven substantially by diacylglycerol and is not solely a dicarbonyl phenomenon; and ROS-mediated injury and 3-deoxyglucosone- and glyoxal-derived adducts operate in parallel with, and can be quantitatively dominant over, the MGO route, in particular tissues and disease stages. What distinguishes MGO is not that it subsumes these mechanisms but that it sits *downstream of several of them simultaneously*; it is generated by all three AGE routes and glycolytic flux, and cleared by a single enzyme system, which is what makes it a useful organizing node for considering shared therapeutic and biomarker strategies. This framing is therefore offered as one lens among several, with the explicit limitation that MGO is neither the sole nor necessarily the dominant mediator in any given complication (Section 9.3).

This paper proceeds as follows. Section 2 examines the four routes of MGO generation. Section 3 examines MGO’s principal protein modification targets. Section 4 examines the relationship between tissue MGO accumulation and the clinical distribution of diabetic complications. Section 5 examines the glyoxalase clearance system and its complementary reductase pathways. Section 6 examines therapeutic strategies. Section 7 examines clinical monitoring approaches with proposed biomarker mappings, framed explicitly as hypothesis-generating. Section 8 compares the proposed framework with existing biomarker strategies. Section 9 discusses limitations and priorities for prospective validation.

## 2. Methylglyoxal Generation: Four Convergent Input Routes

**Figure 1:**
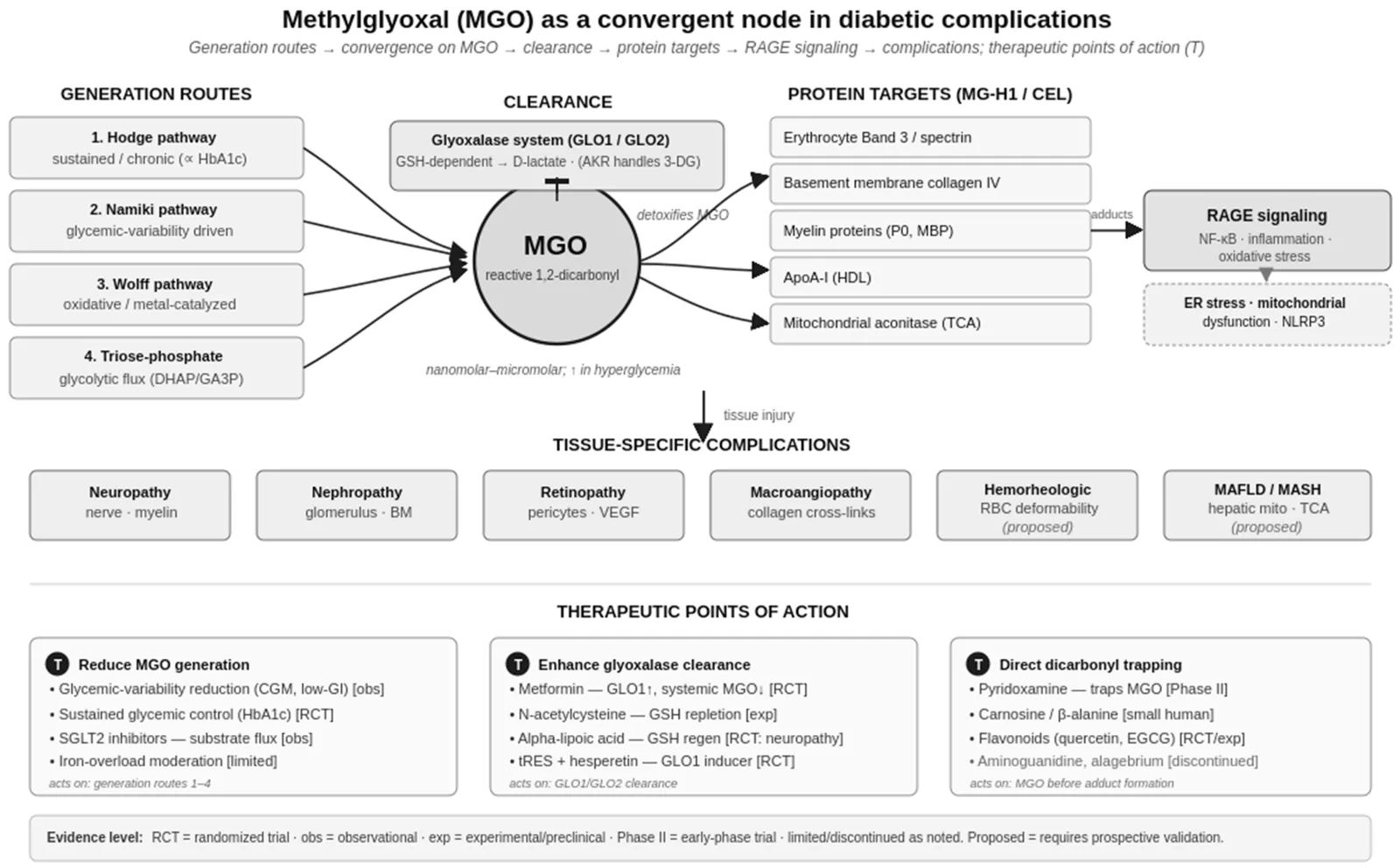
An integrated schematic of methylglyoxal (MGO) generation (four routes), glyoxalase-mediated clearance, tissue-specific protein targets, RAGE signaling, the resulting complications, and the three categories of therapeutic intervention with evidence levels. Arrows indicate the direction of metabolic flow and pathway progression. Entries marked “proposed” are hypothesis-generating and require prospective validation.

In T2DM, MGO is generated at low intracellular concentrations—characteristically in the nanomolar-to-micromolar range, rising within that range (and higher in local microenvironments) under hyperglycemia—through four mechanistically distinct routes. The first three correspond to the classical AGE synthesis pathways; the fourth is a metabolic flux source independent of AGE chemistries. Figure 1 integrates these generation routes with the clearance, protein target, signaling, and therapeutic elements developed across Section 2, Section 3, Section 4, Section 5, Section 6 and Section 7.

### 2.1. The Three AGE Synthesis Routes as MGO Sources

The detailed chemistry of the three classical AGE synthesis pathways has been reviewed elsewhere [4]; here, they are considered specifically as routes of MGO generation, distinguished by the glycemic conditions under which each predominates. The Namiki pathway (retroaldol cleavage of Schiff base intermediates before Amadori rearrangement) has rapid kinetics and is most active under acute glucose flux, generating MGO disproportionately in patients with elevated glycemic variability—including those with controlled mean glucose (HbA1c) but large postprandial excursions [5,6,7]. The Hodge pathway (enolization, dehydration, and retroaldol fragmentation of the stable Amadori product) generates MGO approximately in proportion to time-averaged glucose and is therefore reflected indirectly in HbA1c [3,8,9,10]. The Wolff pathway (transition metal-catalyzed autoxidation of free glucose) yields glyoxal, methylglyoxal, and glucosone and is most active in states of oxidative stress, inflammation, and metal dyshomeostasis that develop progressively in advancing T2DM [11,12,13,14]. The three routes thus differ not in their end product—all converge on MGO—but in the metabolic conditions that drive them, a point central to the convergence argument developed below.

### 2.2. Glycolytic Triose Phosphate Degradation—The Metabolic Flux Source

Independent of the AGE synthesis pathways, MGO is generated continuously through the non-enzymatic decomposition of glycolytic triose phosphate intermediates: dihydroxyacetone phosphate (DHAP) and glyceraldehyde-3-phosphate (GA3P) [15,16]. These intermediates undergo spontaneous β-elimination of phosphate to yield MGO, with the rate proportional to triose phosphate concentration. Under conditions of high glycolytic flux, impaired glyceraldehyde-3-phosphate dehydrogenase activity (as occurs under oxidative or AGE modification stress), or both, triose phosphate concentrations rise and MGO generation through this route can become quantitatively dominant [15,17]. This route is particularly active in tissues with high glycolytic activity such as endothelium and erythrocytes, contributing to the localization of MGO-related damage in these tissues.

### 2.3. Quantitative Considerations

The relative contribution of each route to total cellular MGO varies by tissue, by metabolic state, and by stage of T2DM. Direct quantification studies suggest that under sustained hyperglycemia in vascular tissues, Hodge-derived MGO predominates over chronic timescales; in tissues with high glycolytic flux, the triose phosphate route may equal or exceed Hodge contributions; and under conditions of acute glycemic excursion or oxidative stress, Namiki and Wolff routes contribute disproportionately [3,16,18,19]. These quantitative attributions remain incompletely characterized in vivo and represent an important area for further investigation.

## 3. Methylglyoxal Protein Modification Targets

### 3.1. Reaction Chemistry—Arginine and Lysine Preferences

MGO reacts preferentially with the guanidino group of arginine residues to form methylglyoxal-derived hydroimidazolone-1 (MG-H1), with secondary reactivity at lysine ε-amino groups to form N-ε-carboxyethyl-lysine (CEL) [20,21]. MG-H1 is the most abundant MGO-derived AGE adduct in human tissue, accumulating in a wide range of proteins [9,20,22,23]. Free MG-H1 and free CEL have low intrinsic affinity for RAGE; effective RAGE binding occurs when these adducts are presented as structural components of proteins, including serum albumin, hemoglobin, collagen, and lipoproteins [3,20,24].

In addition to MG-H1 and CEL, MGO contributes to the formation of lysine–lysine cross-links (MOLD) and lysine–arginine cross-links (MODIC) when bis-modification on adjacent residues permits intramolecular or intermolecular bridge formation. These cross-links contribute to the irreversible stiffening of long-lived extracellular matrix proteins observed in advanced T2DM.

### 3.2. Principal Protein Modification Targets Across Tissues

The following protein targets have been identified through proteomic and biochemical analyses as substantively modified by MGO in T2DM. Table 1 presents the current evidence base. Tissue-specific consequences of these modifications, where supported by mechanistic and clinical evidence, are summarized in Section 4.

### 3.3. Downstream Cellular Consequences Beyond Direct Adduct Formation

Beyond covalent modification of individual proteins, MGO and dicarbonyl stress engage several converging stress responses that have received increasing attention. MGO-modified and misfolded proteins contribute to endoplasmic reticulum (ER) stress and activation of the unfolded protein response, linking dicarbonyl load to the broader proteostatic stress of the diabetic state [16,25]. MGO modification of mitochondrial proteins—including TCA cycle enzymes such as aconitate hydratase—contributes to mitochondrial dysfunction and to superoxide overproduction that itself feeds back on dicarbonyl generation, consistent with the mitochondrial-centered unifying mechanism [1,16]. Dicarbonyl stress has also been implicated in NLRP3 inflammasome activation and downstream interleukin-1β/interleukin-18 signaling, providing a mechanistic link between MGO and the low-grade inflammation characteristic of T2DM [25]. These pathways are areas of active investigation and are noted here as emerging extensions of the convergent-node framework rather than as settled mechanisms.

## 4. Tissue Mgo Accumulation and Complication Distribution

The tissue distribution of MGO accumulation in T2DM correlates with the clinical distribution of microvascular and macrovascular complications [3,20,26]. Tissues with the highest documented MGO burdens include the peripheral nerve, the glomerular endothelium and podocytes, retinal pericytes and the endothelium, vascular smooth muscle and intima, erythrocytes, and hepatocyte mitochondria—corresponding to the tissues affected by neuropathy, nephropathy, retinopathy, macroangiopathy, hemorheological impairment, and metabolic-associated fatty liver disease [3].

Three factors converge to produce this tissue-specific vulnerability:

- **Tissue glucose accessibility.** Cells expressing insulin-independent glucose transporters (GLUT1, GLUT3)—including the capillary endothelium, mesangial cells, retinal pericytes, and the peripheral nerve—accumulate intracellular glucose proportional to plasma glucose, providing a substrate for both AGE synthesis pathways and glycolytic triose phosphate flux.
- **Variable GLO1 expression.** GLO1 expression and activity vary substantially across tissues. Polymorphisms in GLO1 (notably the A111E variant) have been associated with altered enzyme activity and modified microvascular complication risk in T2DM cohorts [27,28].
- **Long protein half-lives.** Tissues with long-lived structural proteins—collagen IV in basement membranes (half-life measured in years), myelin proteins, and vascular elastin—accumulate AGE modifications over time, producing cumulative damage even at modest instantaneous MGO concentrations.

Table 2 maps the clinical complications to the proposed primary MGO targets and the routine clinical readouts that may reflect each. We emphasize that the clinical readout mappings in the right column are proposed associations supported by varying levels of mechanistic and epidemiological evidence; we discuss these mappings as hypothesis-generating in Section 7.

## 5. Methylglyoxal Clearance: The Glyoxalase System

### 5.1. GLO1/GLO2 Architecture

Whereas the preceding sections concern MGO production and its protein consequences, cellular MGO exposure is set by the balance between that production and enzymatic removal. The dominant removal route is the glyoxalase couple. In the first and rate-limiting step, glyoxalase I (GLO1) acts on the hemithioacetal that forms spontaneously between MGO and reduced glutathione (GSH), converting it to S-D-lactoylglutathione; glyoxalase II (GLO2) then hydrolyzes that thioester to D-lactate, returning GSH to the free pool [29,30]. The net stoichiometry is therefore MGO detoxified per catalytic cycle with GSH consumed and regenerated rather than net-consumed—a feature that makes GSH availability, rather than its net turnover, the limiting variable (Section 5.3).

An important specificity follows from this architecture: the couple is kinetically tuned to MGO. Glyoxal is processed far less efficiently, and 3-deoxyglucosone is handled not by glyoxalase at all but by aldose reductase and other aldo-keto reductases (AKR family) [30,31]. Clearance of the dicarbonyl pool is thus partitioned—glyoxalase for MGO and reductase systems for the other dicarbonyls—which is why MGO burden in particular can be limited by, or outstrip, glyoxalase capacity independently of the handling of related species.

### 5.2. GLO1 Regulation and Clinical Correlates

GLO1 expression is regulated by Nrf2-dependent transcriptional programs and modulated by AMPK signaling [32]. Both pathways are activated by metformin and several other interventions discussed in Section 6, providing a molecular basis for the pleiotropic anti-AGE effects of these agents independent of their primary glucose-lowering mechanisms.

GLO1 polymorphisms (notably the A111E variant resulting from a single-nucleotide substitution in the GLO1 gene) have been associated with altered enzyme activity in some studies and with modified microvascular complication risk in T2DM cohorts [27]. The clinical translational status of GLO1 polymorphism testing remains investigational; assays for direct measurement of erythrocyte GLO1 activity are available in research settings but are not part of routine clinical practice.

### 5.3. GSH Dependence and Systemic Implications

Because GLO1 requires GSH as a cofactor (with regeneration by GLO2), GSH depletion functionally limits glyoxalase capacity [29]. GSH consumption by glyoxalase clearance, by Phase II xenobiotic detoxification, and by direct ROS neutralization places demand on the cysteine pool. Hepatic GSH turnover under sustained dicarbonyl load may be reflected in markers of glutathione metabolism—including gamma-glutamyl transferase (GGT), which catalyzes the first step in extracellular glutathione recycling—though the relationship between GGT and glyoxalase system load is mechanistically plausible rather than directly validated as a biomarker correlation. This proposed relationship is discussed further in Section 7 [33,34,35].

## 6. Therapeutic Reduction in Methylglyoxal Burden

Therapeutic interventions affecting MGO biology can be grouped into three categories: reduction in MGO generation, enhancement in MGO clearance, and direct dicarbonyl trapping. Within each category, the evidence base for individual interventions varies substantially in quality, and this variation is important: mechanistic plausibility and biomarker-level effects are common, whereas demonstrated benefit on hard clinical endpoints is rare. To make this gradient explicit, each intervention below is annotated with its highest current level of evidence—in vitro, animal, observational, randomized controlled trial (RCT), early-phase (Phase II), or regulatory-approved—and, where relevant, its negative, null, or discontinued findings. We characterize each intervention with reference to this evidence base rather than implying comparative superiority; direct comparative trials between MGO-targeting and other anti-AGE strategies have generally not been performed, and no MGO-targeting strategy has demonstrated hard endpoint superiority over individual pathway interventions.

### 6.1. Reducing MGO Generation

- **Glycemic variability reduction.** *[Evidence: observational/mechanistic.]* Interventions that reduce postprandial glycemic excursions—including time-in-range optimization with continuous glucose monitoring, low-glycemic-index dietary patterns, and GLP-1 receptor agonists with gastric-emptying effects—should reduce Namiki pathway substrate availability for MGO generation [36].
- **Sustained glycemic control.** *[Evidence: RCT—landmark glycemic control trials.]* A reduction in mean glucose exposure (reflected in HbA1c) reduces Amadori product accumulation and proportionally reduces Hodge pathway MGO generation, consistent with the long-term complication–reduction benefit demonstrated in landmark glycemic intervention trials [37,38].
- **SGLT2 inhibitors.** *[Evidence: observational/mechanistic for the MGO attribution.]* By promoting renal glucose excretion and reducing intrahepatic glucose flux, SGLT2 inhibitors reduce substrate availability across multiple MGO-generating routes. Hepatic GGT reduction observed with these agents is consistent with a reduction in glyoxalase system load, though the mechanistic attribution remains hypothesis-generating [39].
- **Iron metabolism moderation.** *[Evidence: limited; indirect.]* Avoiding excess heme iron intake, and treating iron overload where present, limits Wolff pathway Fenton chemistry and the associated oxidative dicarbonyl generation. The clinical evidence for iron moderation-specific outcomes in T2DM is limited [40].

### 6.2. Enhancing Glyoxalase-Mediated Clearance

- **Metformin.** *[Evidence: human—reduces systemic MGO in T2DM; RCT-supported glucose lowering.]* Beyond its primary glucose-lowering effect via AMPK activation, metformin has been demonstrated to upregulate GLO1 expression and to reduce systemic methylglyoxal levels in patients with T2DM, providing a pleiotropic anti-MGO mechanism additive to glucose lowering [41,42].
- **N-acetylcysteine (NAC).** *[Evidence: experimental; limited human data for AGE endpoints.]* NAC provides cysteine as a rate-limiting substrate for de novo GSH synthesis, replenishing the GLO1 cofactor pool. NAC has reduced plasma MGO in experimental models; clinical efficacy data for AGE-related endpoints in T2DM are limited [43].
- **Alpha-lipoic acid.** *[Evidence: RCT for neuropathy symptoms; MGO biomarker effect in humans less documented.]* It acts both as a direct antioxidant and as a GSH regenerator (reducing GSSG to GSH via thioredoxin reductase coupling). It has demonstrated symptomatic improvement in diabetic peripheral neuropathy in randomized trials; its effects on MGO biomarkers in humans are less directly documented [44].

### 6.3. Direct Dicarbonyl Trapping

- **Pyridoxamine.** *[Evidence: Phase II—reduced urinary MG-H1; renal endpoints mixed.]* This is a vitamin B6 analog that directly traps reactive 1,2-dicarbonyls including MGO and GO. Phase II clinical trials in early diabetic nephropathy demonstrated reductions in urinary MG-H1 and improvements in some renal function endpoints [45].
- **Carnosine and β-alanine.** *[Evidence: small human studies; at the surrogate level.]* Carnosine (β-alanyl-L-histidine) reacts with MGO at its imidazole group. β-alanine is the rate-limiting precursor for tissue carnosine synthesis and dietary carnosine is present in red meat and poultry. Modest reductions in plasma MGO have been reported in small human studies [46].
- **Bioflavonoids (quercetin, hesperidin, EGCG).** *[Evidence: RCT/experimental; at the surrogate level.]* Quercetin has reduced plasma methylglyoxal in randomized placebo-controlled trials in healthy adults; hesperidin and related flavonoids show similar in vitro and clinical trial activity. The mechanism involves both direct dicarbonyl trapping and polyphenol-mediated transition metal chelation [47,48,49,50,51].

### 6.4. A Note on AGE-Targeting Agents with Complex Translational Histories

- **Aminoguanidine (pimagedine).** *[Evidence: discontinued—efficacy in animals; human development halted for toxicity.]* This is a hydrazine compound that traps reactive dicarbonyls. It has demonstrated efficacy in animal models of diabetic nephropathy and retinopathy. Clinical development was discontinued following the ACTION-1 trial due to adverse effects including anti-glomerular basement membrane antibody formation and vasculitis. Aminoguanidine remains a research tool and is not in current clinical use [52].
- **Alagebrium (ALT-711).** *[Evidence: discontinued—Phase II compliance signal; development ended.]* This is an AGE cross-link breaker designed to cleave established collagen cross-links. Phase II trials demonstrated improvements in arterial compliance in older adults, but clinical development was discontinued for commercial reasons [53].

These translational histories illustrate the gap between mechanistic rationale and demonstrated clinical efficacy in the AGE-targeting space. The strategies proposed in this paper are therefore framed in terms of mechanistic plausibility and the current evidence base, not in terms of superiority over alternatives that have not been compared in direct trials.

## 7. Clinical Monitoring Approaches—A Hypothesis-Generating Schema

### 7.1. Purpose and Explicit Caveats

Direct measurement of MGO and its principal protein adducts is technically feasible but is not generally available in routine clinical practice. Plasma MGO measurement requires liquid chromatography–tandem mass spectrometry (LC-MS/MS) or stable-isotope dilution assays available only in specialized research laboratories. Urinary MG-H1 measurement by ELISA is more accessible but lacks standardization across laboratories. Skin autofluorescence measurement, which integrates pentosidine and related fluorescent AGEs in collagen, provides a non-invasive indirect measure but is not yet incorporated into routine care.

We present below a hypothesis-generating organizing schema linking routine laboratory parameters—available on standard CBC and CMP—to proposed components of MGO biology (Table 3). We emphasize the following caveats:

- **This schema is hypothesis-generating, not validated.** The proposed mappings between routine biomarkers and MGO-specific biology have a mechanistic rationale developed below but have not been prospectively validated in clinical cohorts. They are presented as testable hypotheses, not as clinical scoring instruments.
- **Each proposed mapping has alternative explanations.** Elevated GGT and elevated RDW each have multiple potential interpretations beyond the MGO-related mechanisms proposed here. The schema should not be applied clinically without acknowledgement of these alternatives.
- **Validation will require prospective biomarker studies.** The validation work needed to convert these proposed mappings into clinically actionable markers is outlined in Section 9.

**Table 3:** The proposed clinical proxies for MGO biology (hypothesis-generating; none validated for clinical use). The GGT and RDW rows in particular are unvalidated proposals discussed in Section 7; all entries are presented with advantages, limitations, availability, and current (research-only) applicability.

Proposed Proxy	MGO-Related Component	Mechanistic Rationale	Caveats/Alternatives
Plasma MGO (LC-MS/MS)	Direct measurement of circulating MGO	Direct biomarker; integrates generation from all four routes	Not available in routine clinical care; requires specialized laboratory
Urinary MG-H1 (ELISA)	Integrated whole-body MG-H1 generation	Stable end product of MGO–arginine reaction; renal excretion correlates with systemic generation	Interlaboratory variability; not standardized; affected by renal function
Skin autofluorescence	Tissue-accumulated AGEs (pentosidine, CML, MG-H1)	Non-invasive measure of long-term tissue AGE burden	Integrates multiple AGE pathways, not MGO-specific; assay standardization developing
GGT (proposed proxy; not validated)	Putative marker of glyoxalase system load	GGT catalyzes extracellular GSH recycling; hepatic GSH turnover under MGO load mechanistically plausible	Multiple confounders (hepatic disease, alcohol, medications); not MGO-specific; mechanistic attribution requires validation
RDW (proposed proxy; not validated)	Putative marker of erythrocyte membrane MGO modification	Band 3/spectrin MG-H1 modification may alter erythrocyte deformability and volume distribution	Multiple confounders (nutritional deficiency, inflammation, ineffective erythropoiesis); not MGO-specific; mechanistic attribution requires validation
HbA1c	Integrated Hodge pathway activity	Amadori product on hemoglobin; reflects sustained glycemic exposure	Reflects only Hodge pathway component; does not capture Namiki/Wolff/triose phosphate contributions to MGO
CGM-derived variability indices	Proxy for Namiki pathway substrate availability	Postprandial excursions drive Schiff base turnover and retroaldol cleavage	Requires CGM availability; variability metrics not yet standardized for clinical decisions

### 7.2. Proposed Clinical Proxies for MGO Biology

The GGT and RDW mappings deserve particular attention, and it is important to be explicit that neither the clinical associations they rest on nor the parameters themselves are original to this manuscript. Both have established observational research in diabetes and metabolic disease. Elevated GGT is a well-documented predictor of incident diabetes, metabolic syndrome, and cardiovascular risk, and has been proposed as a marker of cellular antioxidant (glutathione) inadequacy and oxidative stress [33,35,54,55]. Elevated RDW has been repeatedly associated with glycation and microvascular disease in T2DM—with HbA1c elevation specifically (rather than with glucose per se) [56], with diabetic nephropathy, retinopathy, and vascular dysfunction [57,58,59], with renal tubular injury [60], and with glycemic and lipid indices [61]—and these associations have been summarized in a systematic review and meta-analysis [62]. The only incremental step taken here is interpretive: to consider whether these established associations might, in part, reflect MGO-related biology (glyoxalase system glutathione demand for GGT; erythrocyte membrane MG-H1 modification for RDW). That interpretation is a hypothesis, not a finding. Both proposed mappings require prospective validation before any clinical application, and until such validation exists, GGT and RDW should be interpreted with their established clinical meanings and their numerous confounders (for GGT: hepatic disease, alcohol, medications; for RDW: nutritional deficiency, inflammation, ineffective erythropoiesis, reticulocytosis).

### 7.3. Distinction from a Validated Clinical Scoring Instrument

The schema above is an organizing framework for considering routine biomarkers in the context of MGO biology. It is not a weighted composite score, a threshold-based diagnostic instrument, or a therapeutic decision algorithm. The development of any such instrument would require independent prospective validation in adequately powered cohorts, which is outside the scope of the present mechanistic synthesis.

## 8. Comparison with Existing Diagnostic and Biomarker Strategies

### 8.1. Glycemic Monitoring (HbA1c, Fasting Glucose, CGM)

HbA1c remains the cornerstone of glycemic monitoring, with extensive validation as a predictor of microvascular complications [37,38]. The schema developed in this paper does not replace HbA1c monitoring; it adds putative additional information about pathway-specific MGO contributions that are not captured by HbA1c—particularly the Namiki (variability-driven) and Wolff (oxidative milieu-driven) components. CGM-derived variability indices complement HbA1c by capturing glycemic variability; their interpretation in terms of MGO biology is a hypothesis-generating extension developed here.

### 8.2. Direct AGE Measurements

Direct measurement of AGE adducts—serum CML, plasma MGO, urinary MG-H1, and skin autofluorescence—is feasible but limited to research and specialty clinical settings [25,63]. The advantage of the schema in Section 7 is the use of biomarkers already universally available; the disadvantage is that none of the proposed proxies measure MGO chemistry directly.

### 8.3. GLO1 Activity Assays

Direct measurement of GLO1 activity in erythrocyte lysates is technically feasible but is not part of routine clinical practice [30]. GLO1 polymorphism testing remains investigational. The proposed indirect monitoring of glyoxalase system load through GGT, while substantially less precise than direct GLO1 measurement, has the practical advantage of being available on every routine CMP.

### 8.4. Insulin Resistance Indices

HOMA-IR, the Matsuda Index, and the triglyceride–glucose ratio quantify the systemic milieu in which MGO biology operates: insulin-resistant, pro-inflammatory, and often iron-repleted states are conducive to dicarbonyl generation [64,65]. These indices are complementary to, not redundant with, the pathway-specific biomarkers proposed in Section 7.

## 9. Limitations and Prospective Validation

### 9.1. Mechanistic Claims Rest Predominantly on In Vitro and Animal Model Evidence

The biochemistry of MGO generation, glyoxalase clearance, and MGO–protein adduct formation has been characterized predominantly in cell culture, biochemical kinetics studies, and animal models. The quantitative contribution of each generation route in vivo in human T2DM at specific stages of disease has not been definitively established. Direct quantification of MGO-mediated complication causation—as opposed to correlation—remains an active area of investigation.

### 9.2. The Biomarker Mappings in Section 7 Require Prospective Validation

The proposed mappings of GGT and RDW to MGO-related biology have a defensible mechanistic rationale but have not been prospectively validated in clinical cohorts. Studies linking GGT and RDW trajectories to direct measurements of plasma MGO, urinary MG-H1, and clinical complication endpoints are needed before these mappings can be considered clinically actionable. We make no claim that the schema constitutes a clinical scoring instrument.

### 9.3. The Convergent Mediator Framing Is a Hypothesis-Generating Organizing Schema

The characterization of MGO as a convergent mediator of diabetic complications is an organizing framework synthesizing established dicarbonyl chemistry, glyoxalase biology, and AGE–protein adduct research. It is not a claim that MGO is the sole or dominant mediator of all diabetic complications; tissue-specific contributions of glyoxal, 3-deoxyglucosone, ROS-derived AGE adducts, polyol pathway flux, PKC activation, and hexosamine pathway activity remain biologically important and may be quantitatively dominant in particular settings.

**Scope—type 2 versus type 1 diabetes.** The framework is developed for, and its biomarker proposals are calibrated to, type 2 diabetes mellitus, in which insulin resistance, elevated glycolytic triose phosphate flux, and an iron-repleted, pro-oxidant, inflammatory milieu jointly favor dicarbonyl generation. The core dicarbonyl chemistry is not diabetes type-specific; hyperglycemia-driven MGO generation and glyoxalase clearance operate in type 1 diabetes as well, so the mechanistic account is expected to apply broadly. However, the metabolic context differs (the insulin resistance-associated flux and oxidative milieu emphasized here are features of T2DM), and the observational biomarker literature invoked for the GGT and RDW proposals derives predominantly from T2DM populations. Extrapolation to T1DM should therefore be made cautiously and would require separate validation; broad statements about “diabetes” in this manuscript should be read as referring to T2DM unless otherwise specified.

### 9.4. Therapeutic Claims Are Limited by Absence of Direct Comparative Trials

The argument that MGO-targeting interventions should affect multiple pathway outputs simultaneously is mechanistically sound but does not constitute evidence of clinical superiority over individual pathway interventions. The convergence architecture provides a rationale for comparative studies; it does not substitute for them. The translational histories of aminoguanidine and alagebrium (Section 6.4) illustrate the gap between mechanistic rationale and demonstrated clinical benefit in this space.

### 9.5. Priorities for Prospective Validation

The validation work most likely to convert the synthesis presented here from hypothesis-generating to clinically applicable includes the following:

- **MGO-specific biomarker cohorts:** prospective longitudinal measurement of plasma MGO, urinary MG-H1, GGT, RDW, HbA1c, and CGM-derived variability in defined T2DM populations stratified by disease duration, with correlation to clinical complication endpoints.
- **Pharmacodynamic studies:** evaluation of biomarker responses (plasma MGO, urinary MG-H1, GGT, RDW) to interventions with established or hypothesized glyoxalase-targeting effects (metformin, NAC, pyridoxamine, β-alanine) to establish target engagement readouts.
- **Comparative therapeutic trials:** head-to-head evaluation of MGO-targeting versus individual pathway interventions, with hard clinical endpoints (eGFR, monofilament, retinopathy progression).
- **Assay standardization:** interlaboratory standardization of plasma MGO and urinary MG-H1 measurement to support multicenter validation studies.

## 10. Conclusions

Methylglyoxal occupies a convergent position in the biochemistry of diabetic complications. Generated by all three classical AGE synthesis pathways (Hodge, Namiki, Wolff) and additionally by the non-enzymatic degradation of glycolytic triose phosphate intermediates, cleared predominantly by the GLO1/GLO2 glyoxalase system (with complementary AKR family reductase handling of related dicarbonyls), and producing tissue damage through a conserved pattern of arginine guanidino and lysine ε-amino modification, MGO is a mechanistically prominent node in the network of biochemical processes linking hyperglycemia to clinical complication.

The convergence chemistry has implications for therapeutic strategy—favoring interventions on shared mediators (MGO generation reduction, glyoxalase system support, direct dicarbonyl trapping) over interventions targeting individual upstream pathways—and for the design of clinical biomarker panels. We have proposed a hypothesis-generating organizing schema linking accessible routine biomarkers to MGO biology, with explicit acknowledgement that the proposed mappings (particularly the GGT and RDW associations) require prospective validation before clinical application.

The synthesis offered here is not the discovery of MGO’s biochemical roles, which have been characterized in the dicarbonyl chemistry and glyoxalase literature for several decades. It is the integration of these mechanistic elements into a clinically oriented framework that addresses a practical question: in the routine biomarker landscape available to the practicing clinician, what may be reasonably interpreted in terms of MGO biology? The framework proposed is offered as a starting point for that question, not as a settled answer.

## Figures and Tables

**Table 1:** Principal MGO protein modification targets identified in T2DM.

Protein Target	Tissue Compartment	Modification Type	Documented Functional Consequence
Band 3/spectrin	Erythrocyte membrane	MG-H1 on multiple Arg residues	Altered cell deformability; impaired capillary transit
ApoA-I (HDL)	Plasma/vascular wall	MG-H1 on Arg173, Arg149	Reduced ABCA1-mediated cholesterol efflux; impaired HDL function
Collagen IV/laminin	Glomerular and vascular basement membrane	MG-H1 + MOLD cross-links	Basement membrane thickening; altered charge selectivity
Myelin proteins (P0, MBP)	Peripheral nerve	MG-H1 on multiple Arg residues	Myelin structural disruption; slowed conduction velocity
Mitochondrial aconitase	All tissues with active TCA	MG-H1 at Arg active site	Reduced enzymatic activity; citrate accumulation
VEGF receptor (extracellular domain)	Retinal endothelium/pericytes	MG-H1 modification of extracellular Arg sites	Modified VEGF signaling response

*Evidence note for Table 1.* The protein modifications themselves (e.g., MG-H1 on erythrocyte Band 3/spectrin, on ApoA-I, on basement membrane collagen, and on mitochondrial aconitase) are well documented in the proteomic and biochemical literature and may be read as established. By contrast, several modification-to-clinical consequence mappings are inferential to varying degrees: the erythrocyte deformability and VEGF-signaling consequences in particular rest on partial mechanistic evidence, whereas the collagen and ApoA-I functional consequences are better supported. Readers should interpret the “documented functional consequence” column accordingly; some links remain hypothesized rather than demonstrated [20,25].

**Table 2:** The proposed MGO-mediated mechanisms for diabetic complications (a conceptual model requiring prospective validation). The “proposed primary MGO mechanism” and “proposed accessible biomarker” columns represent hypothesized associations, not clinically established causal relationships; the biomarker mappings are discussed as hypothesis-generating in Section 7.

Complication	Tissue Compartment	Proposed Primary MGO Mechanism	Established Clinical Readout	Proposed Accessible Biomarker
Peripheral neuropathy	Peripheral nerve	MG-H1 disruption of myelin proteins and tubulin	Monofilament exam	Plasma MGO; urinary MG-H1
Nephropathy	Glomerulus	Basement membrane MG-H1 cross-links; podocyte MGO accumulation	ACR; eGFR	Urinary MG-H1
Retinopathy	Retinal endothelium/pericytes	Pericyte loss; modified VEGF signaling	Fundoscopy	Plasma MGO
Macroangiopathy	Vascular wall	MOLD cross-links in collagen/elastin; ApoA-I modification	Pulse wave velocity; TG/HDL	Skin autofluorescence
Hemorheological impairment (proposed)	Erythrocyte membrane	Band 3/spectrin MG-H1 modification (proposed mechanism)	Not routinely measured	RDW (proposed marker; requires validation)
MAFLD/MASH progression (proposed)	Hepatocyte mitochondria	Aconitase MG-H1; TCA cycle impairment (proposed mechanism)	ALT; ultrasound; FIB-4	GGT (proposed marker; requires validation)

## Data Availability

No new data were created or analyzed in this study. Data sharing is not applicable to this article.

## Abbreviations

3-DG, 3-deoxyglucosone; ABCA1, ATP-binding cassette transporter A1; ACR, albumin-to-creatinine ratio; AGE, advanced glycation end product; AKR, aldo-keto reductase; ALT, alanine aminotransferase; AMPK, AMP-activated protein kinase; ApoA-I, apolipoprotein A-I; CEL, N-ε-carboxyethyl-lysine; CGM, continuous glucose monitoring; CML, N-ε-carboxymethyl-lysine; CMP, comprehensive metabolic panel; DHAP, dihydroxyacetone phosphate; eGFR, estimated glomerular filtration rate; ELISA, enzyme-linked immunosorbent assay; ER, endoplasmic reticulum; FIB-4, Fibrosis-4 index; GA3P, glyceraldehyde-3-phosphate; GGT, gamma-glutamyl transferase; GLO1, glyoxalase I; GLO2, glyoxalase II; GO, glyoxal; GSH, reduced glutathione; GSSG, oxidized glutathione; HbA1c, glycated hemoglobin; HDL, high-density lipoprotein; HOMA-IR, homeostatic model assessment of insulin resistance; LC-MS/MS, liquid chromatography–tandem mass spectrometry; MAFLD, metabolic-associated fatty liver disease; MASH, metabolic dysfunction-associated steatohepatitis; MBP, myelin basic protein; MGO, methylglyoxal; MG-H1, methylglyoxal-derived hydroimidazolone-1; MODIC, methylglyoxal-derived lysine–arginine cross-link; MOLD, methylglyoxal-derived lysine–lysine cross-link; NAC, N-acetylcysteine; NLRP3, NLR family pyrin domain-containing 3; Nrf2, nuclear factor erythroid 2-related factor 2; RAGE, receptor for advanced glycation end products; RDW, red cell distribution width; ROS, reactive oxygen species; SGLT2, sodium–glucose cotransporter 2; T1DM, type 1 diabetes mellitus; T2DM, type 2 diabetes mellitus; TCA, tricarboxylic acid (cycle); TG, triglyceride; VEGF, vascular endothelial growth factor.
